# A Neuroprotective Locus Modulates Ischemic Stroke Infarction Independent of Collateral Vessel Anatomy

**DOI:** 10.3389/fnins.2021.705160

**Published:** 2021-08-02

**Authors:** Han Kyu Lee, Sarah E. Wetzel-Strong, David L. Aylor, Douglas A. Marchuk

**Affiliations:** ^1^Department of Molecular Genetics and Microbiology, Duke University Medical Center, Durham, NC, United States; ^2^Department of Biological Sciences, North Carolina State University, Raleigh, NC, United States

**Keywords:** ischemic stroke, infarct volume, neuroprotection, wild-derived mouse strain, quantitative trait locus mapping

## Abstract

Although studies with inbred strains of mice have shown that infarct size is largely determined by the extent of collateral vessel connections between arteries in the brain that enable reperfusion of the ischemic territory, we have identified strain pairs that do not vary in this vascular phenotype, but which nonetheless exhibit large differences in infarct size. In this study we performed quantitative trait locus (QTL) mapping in mice from an intercross between two such strains, WSB/EiJ (WSB) and C57BL/6J (B6). This QTL mapping revealed only one neuroprotective locus on Chromosome 8 (Chr 8) that co-localizes with a neuroprotective locus we mapped previously from F2 progeny between C3H/HeJ (C3H) and B6. The allele-specific phenotypic effect on infarct volume at the genetic region identified by these two independent mappings was in the opposite direction of the parental strain phenotype; namely, the B6 allele conferred increased susceptibility to ischemic infarction. Through two reciprocal congenic mouse lines with either the C3H or B6 background at the Chr 8 locus, we verified the neuroprotective effects of this genetic region that modulates infarct volume without any effect on the collateral vasculature. Additionally, we surveyed non-synonymous coding SNPs and performed RNA-sequencing analysis to identify potential candidate genes within the genetic interval. Through these approaches, we suggest new genes for future mechanistic studies of infarction following ischemic stroke, which may represent novel gene/protein targets for therapeutic development.

## Introduction

Ischemic stroke is caused by a disruption of the blood supply to the brain leading to neuronal cell death in the ischemic region. Annually, fifteen million people worldwide suffer a stroke (Bogousslavsky et al., 2003; Johnson et al., 2016; WHO, 2017), resulting in an estimated $65.5 billion in healthcare costs per year (Benjamin et al., 2019). In the United States, stroke is the fourth-leading cause of death with almost 800,000 new or recurrent cases occurring each year (Roger et al., 2012). Risk factors for stroke include environmental influences (e.g., smoking, diabetes, obesity, etc.), but epidemiologic studies have estimated that two-thirds of the risk for developing ischemic stroke is due to genetic factors (Jamrozik et al., 1994; Dichgans, 2007). GWAS for ischemic stroke, including a recent meta analysis, have identified dozens of variants in genes involved in diverse biological processes, and many of these variants share risk factors with other vascular traits or intermediate phenotypes of stroke (Malik et al., 2018). Pharmacological interventions to reduce stroke risk have focused on reducing these related vascular risk factors (Malik et al., 2018).

By contrast, once a stroke has occurred, the only approved drug for stroke therapy is intravenous recombinant tissue plasminogen activator (tPA), currently given to only 2–3% of stroke patients because of its limited time window for administration and the major risk for adverse effects. Unfortunately, tPA does not provide protection to neural tissues already damaged by stroke. Thus, there is an urgent need to identify and develop new drug targets to provide effective neuroprotection and/or neuroresuscitation to damaged tissues. Identifying such gene targets has been problematic. In contrast to the successes of genetic studies of stroke risk, GWAS of stroke outcomes among ischemic stroke patients are intrinsically difficult due to uncontrollable variation in the extent and location of the occluded vessel, strong environmental variables (e.g., diet, smoking, etc.), and especially variation in the critical time between first recognized symptoms of stroke and medical intervention.

We have attempted to identify genes that influence infarct volume, the critical outcome of ischemic stroke, by using a surgically induced mouse model of ischemic stroke and subsequent QTL mapping analysis. We have identified several genetic loci that regulate infarct volume (Keum and Marchuk, 2009; Chu et al., 2013; Keum et al., 2013; Lee et al., 2019) and identified several genes modulating this trait (Keum et al., 2013; Lee et al., 2016, 2018). The strongest locus controlling infarct volume in mice (Keum et al., 2013) also modulates the collateral vascular anatomy (Wang et al., 2012). Both traits are highly heritable across strains of mice. Heritability (*H*^2^) for infarct volume was calculated at 88% (Keum and Marchuk, 2009) and for collateral vessel number at 85% (Zhang et al., 2010). The collateral circulation enables vascular reperfusion of the ischemic territory, thus diminishing the effects of ischemia. However, as the collateral vessel anatomy is established early in life due to genetic factors (Chu et al., 2013), we sought to identify genes that modulate infarct volume independently of the collateral circulation in the hopes of identifying loci and genes that modulate infarct size through an innate, neuroprotective mechanism. Our approach for identifying neuroprotective loci was to perform QTL mapping in crosses between inbred mouse strains that exhibit similar collateral vessel anatomy, but which nonetheless show large differences in infarct volume after ischemic stroke. In our survey of strain phenotypes, we included the eight founder mouse strains of the collaborative cross (CC). The CC includes three wild-derived strains, CAST/EiJ (CAST), PWK/PhJ (PWK), and WSB, which contain extensive diversity compared to classical inbred mouse strains (Complex Trait Consortium, 2004; Threadgill, 2006; Aylor et al., 2011). Candidate genes mapping within linkage peaks are identified through shared ancestral haplotype analysis, strain-specific RNA expression analysis, and *in silico* analysis of coding variants. Highly compelling candidate genes are then validated with phenotypic studies using genetically modified mice. Through this approach, we have identified loci and genes that modulate infarct volume independent of the effects of the collateral vasculature (Chu et al., 2013; Keum et al., 2013; Lee et al., 2016, 2018, 2019).

In the present study, by performing QTL mapping between a wild-derived inbred strain, WSB, and a common classical inbred strain, B6, we identified a neuroprotective locus that overlaps with a locus mapped in an independent QTL analysis between two classical inbred strains, C3H and B6. Using reciprocal congenic mouse lines, we validate that this locus modulates infarct volume in a collateral-independent manner. By examining non-synonymous coding SNP variation and strain-specific differential gene expression, we suggest potential candidate genes within the locus.

## Materials and Methods

### Animals

All inbred mouse strains were obtained from the Jackson Laboratory (Bar Harbor, ME, United States), and then bred locally to obtain mice used in all experiments. We generated reciprocal congenic mouse lines in order to evaluate the *Civq4* QTL effect on both genetic backgrounds. (B6 × C3H) F1 animals were backcrossed to B6 to generate B6.C3H-*Civq4* congenics, which we refer to as LineB. (B6 × C3H) F1 animals were backcrossed to C3H to generate C3H.B6-*Civq4*, which we refer to as LineC. Briefly, F1 animals were backcrossed to either B6 or C3H for 4 generations and then the genetic background of each animal was determined by whole genome SNP genotyping (OpenArray Technology). Female mice that contained over 90% of the recipient genetic background were genotyped with additional SNP and microsatellite markers to confirm retention of the *Civq4* region from the relevant strain. These mice were then backcrossed to the opposite strain for 7–10 generations before brother-sister mating to create the congenic lines in the study. Mice (both male and female animals) were age-matched (P21 for collateral vessel density and 12 ± 1 week for pMCAO) for all experiments.

### Collateral Vessel Density Measurement

Since collateral vessels are established by 3 weeks of age and remain constant for many months (Chu et al., 2013), the collateral vessel phenotype was measured at P21 as previously described (Lee et al., 2016, 2018). Briefly, mice were anesthetized with ketamine (100 mg/kg) and xylazine (2.5 mg/kg), and the ascending thoracic aorta was cannulated. The animals were perfused with freshly made buffer (1 mg/ml adenosine, 40 g/ml papaverine, and 25 mg/ml heparin in PBS) to remove the blood. The pial circulation was then exposed after removal of the dorsal calvarium and adherent dura mater. The cardiac left ventricle was cannulated and a polyurethane solution with a viscosity sufficient to minimize capillary transit (1:1 resin to 2-butanone, PU4ii, and VasQtec) was slowly infused; the cerebral circulation was visualized under a stereomicroscope during infusion. After the infusion, the brain surface was topically rinsed with 10% PBS-buffered formalin and the dye solidified for 20 min. After post-fixation with 10% PBS-buffered formalin, the pial circulation was imaged. All collaterals interconnecting the anterior cerebral artery (ACA) and middle cerebral artery (MCA) trees of both hemispheres were counted.

### Permanent Middle Cerebral Artery Occlusion (pMCAO)

Focal cerebral ischemia was induced by direct permanent occlusion of the distal MCA as previously described (Lee et al., 2016, 2018). Briefly, adult mice were anesthetized with ketamine (100 mg/kg) and xylazine (2.5 mg/kg). The right MCA was exposed by a 0.5 cm vertical skin incision midway between the right eye and ear under a dissecting microscope. After the temporalis muscle was split, a 2-mm burr hole was made with a high-speed micro drill at the junction of the zygomatic arch and the squamous bone through the outer surface of the semi-translucent skull. The MCA was clearly visible at the level of the inferior cerebral vein. The inner layer of the skull was removed with fine forceps, and the dura was opened with a 32-gauge needle. While visualizing under an operating microscope, the right MCA was electrocauterized. The cauterized MCA segment was then transected with micro scissors to verify permanent occlusion. The surgical site was closed with 6-0 sterile nylon sutures, and 0.25% bupivacaine was applied. The temperature of each mouse was maintained at 37°C with a heating pad during the surgery until the animal was fully recovered from the anesthetic. Mice were then returned to their cages and allowed free access to food and water in an air-ventilated room with the ambient temperature set to 25°C.

### Infarct Volume Measurement

Cerebral infarct volumes were measured 24 h after surgery because the size of the cortical infarct is largest and stable at 24 h after distal permanent MCA occlusion (Lambertsen et al., 2005). Twenty-four hours after MCAO surgery, the animals were euthanized with CO_2_ inhalation followed by decapitation, and the brains were carefully removed. The brains were placed in a brain matrix and sliced into 1 mm coronal sections after being chilled at −80°C for 4 min to slightly harden the tissue. Each brain slice was placed in 1 well of a 24-well plate and incubated for 20 min in a solution of 2% 2,3,5-triphenyltetrazolium chloride (TTC) in PBS at 37°C in the dark. The sections were then washed once with PBS and fixed with 10% PBS-buffered formalin at 4°C. Then, 24 h after fixation, the caudal face of each section was scanned using a flatbed color scanner. The scanned images were used to determine infarct volume (Wexler et al., 2002). Image-Pro software (Media Cybernetics) was used to calculate the infarcted area of each slice by subtracting the infarcted area of the hemisphere from the non-infarcted area of the hemisphere to minimize error introduced by edema. The total infarct volume was calculated by summing the individual slices from each animal.

### SNP Genotyping

Genomic DNA was isolated from tails of F2 progeny between WSB and B6 mice using DNeasy Tissue kit (Qiagen, Hilden, Germany). Genome-wide SNP genotyping was performed with a new Mouse Universal Genotyping Array (MiniMUGA), an array-based genetic QC platform with over 11,000 probes. Array hybridization including sample preparation was performed by Neogen/GeneSeek (Lincoln, NE, United States).

### Quantitative Trait Locus (QTL) Analysis

Using R/qtl software, genome-wide scans were performed as previously described (Lee et al., 2019) with minor changes. Genotype information from the MiniMUGA were prepared for QTL mappings as followed. A total of 515 informative markers for WSB and B6 across the mouse genome were used for genetic mapping. The significance thresholds for LOD scores were determined by 1,000 permutations using all informative markers. A QTL was considered significant when its LOD score exceeded 95% (*p* < 0.05) of the permutation distribution. The confidence interval of the peak was determined by the 1.5-LOD support interval. The physical map megabase (Mb) positions based on the genomic sequence from the GRCm38/mm10 were calculated using Mouse Map Converter tool of the Jackson Laboratory^1^. For the interval *Civq4*, SNP data were obtained from the Mouse Phenome Database^2^.

### SNP Analysis

The effect of non-synonymous SNPs on protein function was predicted by using three independents *in silico* prediction algorithms, Polymorphism Phenotyping v2 (PolyPhen-2)^3^ (Adzhubei et al., 2010), Sorting Intolerant From Tolerant (SIFT)^4^ (Ng and Henikoff, 2003), and Protein Variation Effect Analyzer (PROVEAN)^5^ (Choi et al., 2012).

### RNA Sequencing Analysis and Differential Gene Expression

Paired-end, 150 bp sequencing reads were generated from adult (8–12 weeks) brain cortex tissue mRNA of B6, C3H, and WSB mice (three male mice for each strain) on the NovaSeq 6000 instrument. Adapters for all paired-end sequencing reads were trimmed using Cutadapt (Martin, 2011) and gene expression counts for each sample were obtained using HTseq (Anders et al., 2015). Differential gene expression analysis between two groups was performed using DESeq2 R package. DESeq2 provides statistical routines for determining differential expression in digital gene expression data using a model based on the negative binominal distribution. *p*-values were adjusted using a false discovery rate of 5% by Benjamini-Hochberg correction (Benjamini and Hochberg, 1995). The RNA sequencing and differential gene expression analyses were performed by Novogene (Sacramento, CA, United States).

### Statistical Analysis

Statistical analyses were performed with Prism (GraphPad Software, La Jolla, CA, United States). Statistical significance was evaluated using one-way ANOVA followed by Tukey’s multiple comparison test, according to the following definition: *p* > 0.05, not significant; *p* < 0.05, significant (^∗^); *p* < 0.01, very significant (^∗∗^); and *p* < 0.001, highly significant (^∗∗∗^).

## Results

### A Locus on Chromosome 8 Regulates Infarct Volume in a Cross Between Two Inbred Mouse Strains, B6 and WSB

We returned to our phenotypic characterization of the eight founder mouse strains of the CC (Lee et al., 2019) in order to identify strains that exhibited differences in infarct volume due to vascular-independent effects. As predicted by the role of collateral vessels in the reperfusion of ischemic tissue, the classical inbred strain B6 exhibits a high number of collateral vessel connections with a correspondingly small infarct volume. Surprisingly, although the wild-derived strain WSB shows an even greater number of collateral vessel connections than B6, WSB mice have much larger infarct volumes. This break between the collateral vessel density and infarct volume phenotypes in WSB compared to B6 motivated the choice of these two strains for a new mapping cross with the goal of identifying additional genetic loci that regulate infarct volume.

**FIGURE 1:**
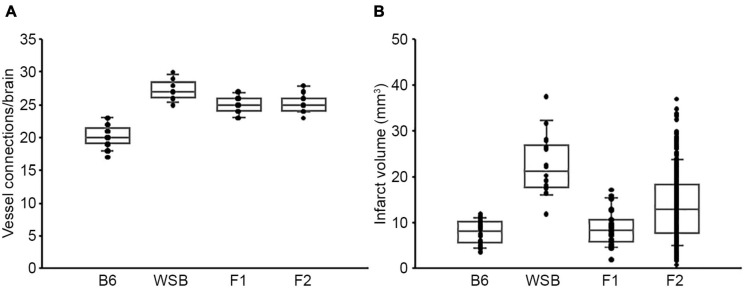
F2 progeny between B6 and WSB display a wide distribution of infarct volume after pMCAO. **(A)** The scatter plot indicates the number of collateral vessel connections between the ACA and MCA for parental strains, B6 and WSB, and their F1 and F2 intercross animals. The box plot shows the degree of dispersion and skewness. The number of animals for B6, WSB, F1, and F2 was 37, 13, 21, and 30 animals, respectively. **(B)** The scatter and box plot graphs show the distribution of infarct volume after pMCAO for B6, WSB, and their F1 and F2 intercross animals. The number of animals for the infarct volume measurements was 34, 18, 27, and 376 animals, respectively.

To discover the genetic region(s) modulating ischemic infarction across these strains, we first generated F1 progeny between the strains, and then performed an intercross. Then, F1 and F2 animals were analyzed for collateral vessel and infarct volume phenotypes. The number of collateral vessel connections among the F1 animals was tightly distributed approximately midway between the parental strains [B6 (20.4), WSB (27.3), F1 (24.9)] (Figure 1A). The infarct volume among the F1 animals was also relatively tightly distributed, but much closer to the low value seen in B6 animals [B6 (7.8 mm^3^), WSB (22.6 mm^3^), F1 (8.9 mm^3^)] (Figure 1B). Collateral vessel number measured in a separate cohort of F2 animals was tightly distributed around the mean of 25.3 (Figure 1A). By contrast, infarct volume in the F2 animals exhibited a very wide distribution, ranging from 0.8 to 37.1 mm^3^ (Figure 1B). The tight distribution of collateral vessel numbers in the F2 animals, contrasted with the wide variation in infarct volume, validated our choice of these strains for our attempt to map collateral-independent loci that modulate infarct size.

**FIGURE 2:**
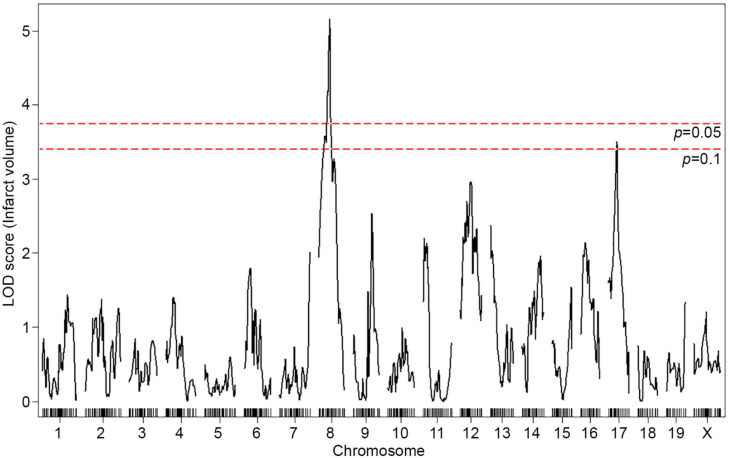
A locus for infarct volume is mapped in the F2 progeny between B6 and WSB. The graph presents the analysis of a genome-wide QTL mapping scan for infarct volume measured 24 h after pMCAO using 376 F2 animals (B6 and WSB). Chromosomes 1 through X are represented numerically on the *x*-axis and the *y*-axis represents the LOD score. The significant (*p* < 0.05) level of linkage was determined by 1,000 permutation tests. Only a single genomic region on Chr 8 displays significant linkage to infarct volume with a LOD score of 5.16.

To map the genetic region(s) that modulate infarct volume in this cross, we performed genome-wide QTL mapping analysis. A total of 376 F2 mice (Figure 1B) were genotyped with a genome-wide mapping panel (the miniMUGA genotyping panel consisting of approximately 11K SNPs). For initial genome-wide QTL mapping analysis, 515 informative SNP markers were selected across the mouse genome (Supplementary Table 1), which completely covered the entire mouse genome at approximately 5 Mb intervals across each chromosome. We identified a QTL peak on Chr 8 (logarithm of the odds, LOD, 5.16; *p* = 0.002) that displayed significant linkage to the infarct volume trait (Figure 2). No other region reached statistical significance at *p* = 0.05, as determined by data permutation, but one peak on Chr 17 reached a LOD of 3.51, corresponding to a suggestive *p*-value of 0.083. These two loci show no evidence of genetic interaction.

The infarct volume phenotype of the F2 animals was relatively normally distributed, although with a tail at the high-end values (Supplementary Figure 1). To determine if deviations from normality were influencing our mapping analysis, we also performed non-parametric mapping. The linkage profiles across the entire genome were nearly identical, including at the linkage peaks on Chr 8 and 17 (Supplementary Figure 2). Thus, the mapping results appear robust to any deviations from normality.

### Characterization of the Location, Size, and Alleleic Effects of the Chromosome 8 Locus

**FIGURE 3:**
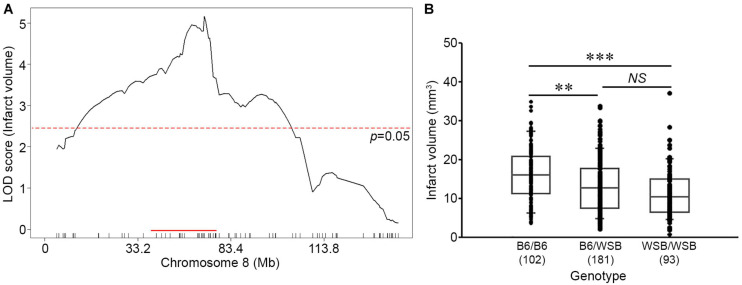
Characterization of the location, size, and allelic effects on infarct volume at the Chromosome 8 locus. **(A)** The graph shows the QTL mapping across Chr 8 using 88 informative SNP markers. The LOD score at the peak is 5.16 (gUNC14975151), and the 1.5-LOD support interval is from 36.16 to 78.11 Mb, indicated by the red bar on the graph. The significance threshold for linkage was calculated using the 88 informative SNP markers on Chr 8. **(B)** Allelic effects on infarct volume of the F2 cohort at gUNC14975151. The B6 allele confers increased susceptibility to infarction and the WSB allele confers protection. Statistical analysis was performed with one-way ANOVA followed by Tukey’s multiple comparison test (^∗∗^*p* < 0.01; ^∗∗∗^*p* < 0.001). NS, not significant.

To fine-map the statistically significant peak mapping to Chr 8, we extended the interval an additional 1.5 Mb flanking each arm of the 1.5-LOD support interval; a convention that defines a conservative estimate of the locus. A total of 88 additional informative SNPs markers were included in the linkage data to more precisely map the locus. The resulting candidate 1.5-LOD interval falls between 36.16 and 78.11 Mb (Figure 3A, Table 1, and Supplementary Table 1).

**TABLE 1:** Characteristics of two independent QTL mappings for surgically induced ischemic infarct volume.

**Location**	**LOD score**	**1.5-LOD interval (Mb)**	**Marker at peak (position, Mb)**	**Mapping strains**	**Protective allele**	**Variance (%)**
Chr 8	5.16	8:36.16-78.11 (41.95 Mb)	gUNC14975151 (70.88 Mb)	B6 and WSB	WSB	11
Chr 8	10.31	8:36.02-50.26 (14.24 Mb)	rs13479735 (45.52 Mb)	B6 and C3H	C3H	31

Surprisingly, the direction of the allele-specific phenotypic effect at this locus was in the opposite direction relative to what we expected based on the parental strain phenotypes. Although infarct volume for B6 mice is smaller than that of WSB mice, we found that among the F2 animals, B6 homozygotes at this locus exhibited larger infarct volumes than WSB homozygotes with heterozygotes animals falling between these values (Figure 3B). Thus, unexpectedly, the WSB allele from this otherwise highly sensitive inbred strain confers resistance to ischemic tissue damage at this locus.

To survey how other loci affect infarct volume, we examined the allelic effects of the next highest peak, mapping to Chr 17. Although this locus did not reach the significance threshold level (Figure 2), B6 homozygotes at this locus exhibit a protective effect on infarct volume, in agreement with the phenotype of the parental B6 strain (Supplementary Figure 3 and Supplementary Table 1). These data suggest that effects from many different regions of the genome, including the Chr 17 locus, drive the overall infarct volume phenotype in the WSB and B6 strains, and override the opposite allelic effects of the locus mapping to Chr 8.

### Concordance With a Previously Mapped QTL

The location and the directional phenotypic effect at the Chr 8 locus parallels those of another infarct volume QTL mapping to mouse Chr 8. Like WSB, the classical inbred mouse strain C3H breaks the inverse correlation between collateral vessel density and ischemic damage, with a relatively high number of collateral vessel connections but showing a paradoxically large infarct volume. Infarct volume in the B6 strain (7.8 mm^3^) is approximately 3-fold smaller than that observed in both C3H (26.4 mm^3^) and WSB (22.6 mm^3^) strains (Supplementary Figure 4). In a cross between C3H and B6, we previously mapped a highly significant QTL to Chr 8, where the direction of the allele-specific phenotypic effect at this locus was opposite to what was observed for the parental strains (Chu et al., 2013). This locus, named *Civq4*, overlaps the locus mapped here between WSB and B6. The wild-derived strain WSB is more distantly related than either of the classical strains, providing a hypothesis that would aid in fine-mapping the locus. If we assume the same QTL is segregating in both crosses, we would expect the ancestral haplotypes to follow the pattern of WSB = C3H ≠ B6 in the candidate region (Kelada et al., 2012; Widmayer et al., 2020).

We thus re-analyzed our previous QTL mapping data between B6 and C3H to provide a robust comparison to the locus mapped in this study. Using 285 informative SNP markers along with the infarct volume phenotype of 210 F2 animals in the cross between B6 and C3H, we performed genome-wide QTL mapping analysis (Supplementary Table 1). Similar to the previous QTL mapping data (Chu et al., 2013), we observed a highly significant linkage peak (LOD 10.31) located on Chr 8 (Supplementary Figure 5A). The 1.5-LOD support interval of *Civq4* (36.02–50.26 Mb) falls almost entirely within the 1.5-LOD support interval of this newly mapped locus (36.16–78.11 Mb) (Figure 3A, Supplementary Figure 5B, Table 1, and Supplementary Table 1). As previously noted (Chu et al., 2013), the allele-specific phenotypic effect of this locus was also opposite to that observed for the parental strains (Supplementary Figure 5C). Thus, in two independent QTL mapping experiments with three different inbred strains, we have identified a genetic region on Chr 8 where the B6 allele confers increased sensitivity to infarction.

### Reciprocal Congenic Mouse Lines Validate the Phenotypic Effects of the QTL Interval on Chromosome 8

**FIGURE 4:**
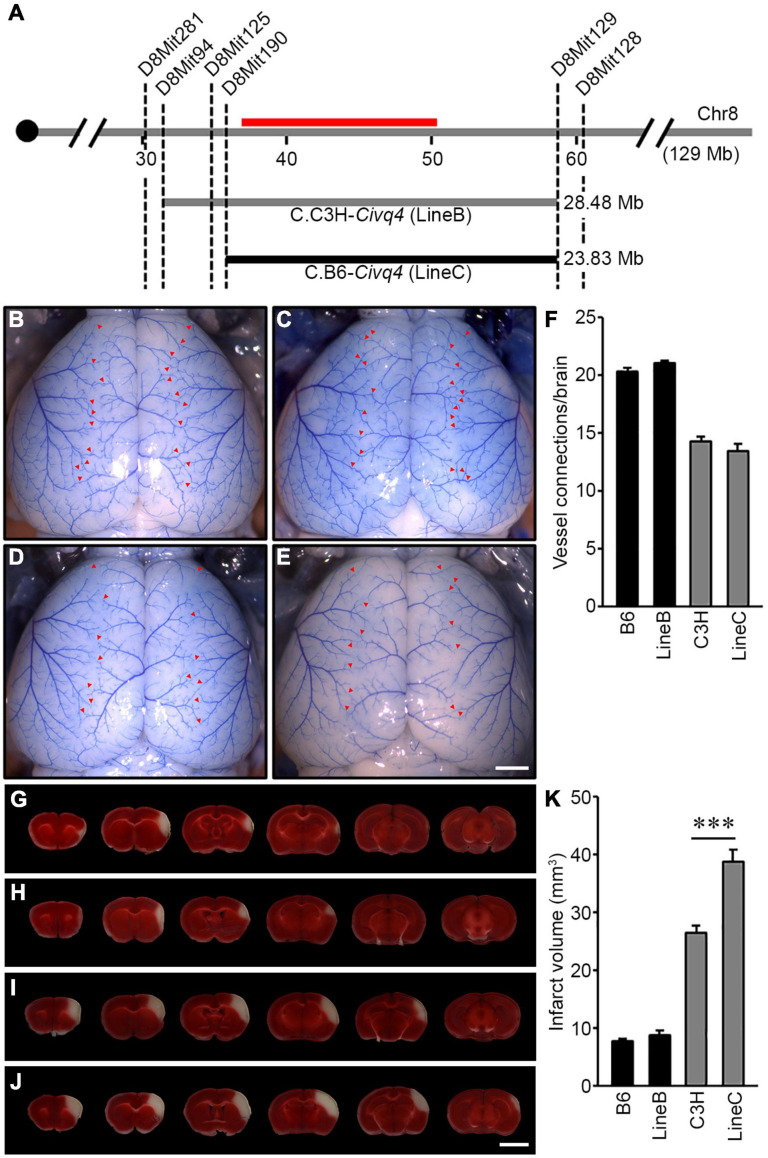
Validation of the location and allelic effects of the locus on Chromosome 8 in congenic animals. **(A)** Schematic of the previously mapped *Civq4* interval using reciprocal congenic mouse lines carrying either a segment of C3H Chr 8 introgressed into the B6 background [B6.C3H-*Civq4* (LineB)] or a segment of B6 Chr 8 introgressed into the C3H background [C3H.B6-*Civq4* (LineC)]. The red bar indicates the 1.5-LOD support interval of QTL mapping between B6 and C3H. Together, these reciprocal congenic mouse lines cover the entire interval of the *Civq4*. **(B–E)** Representative images of the brains for inbred mouse strains, B6 **(B)** and C3H **(D)**, and reciprocal congenic mouse lines, LineB **(C)** and LineC **(E)**. The red arrowheads indicate collateral vessel connections between the ACA and MCA. Scale bar: 1 mm. **(F)** The graph shows the average number of collateral vessel connections between the ACA and MCA in the brain. The total number of animals for B6, LineB, C3H, and LineC were 37, 30, 21, and 12 mice, respectively. Data represent the mean ± SEM. **(G–J)** Serial brain sections (1 mm) for both inbred strains, B6 **(G)** and C3H **(I)**, and the reciprocal congenic mouse lines, LineB **(H)** and LineC **(J)**, are shown. The infarct appears as white tissue after 2% TTC staining. Scale bar: 5 mm. **(K)** The graph indicates the infarct volume 24 h after pMCAO. The total number of animals for B6, LineB, C3H, and LineC were 34, 35, 24, and 16 mice, respectively. Data represent the mean ± SEM. Statistical analysis was performed with one-way ANOVA followed by Tukey’s multiple comparison test (***p < 0.001).

To validate the phenotypic effects of the previously mapped *Civq4* locus, we generated reciprocal congenic mouse lines carrying a segment of the *Civq4* region either from the B6 introgressed into the C3H background (C3H.B6-*Civq4*; LineC) or from the C3H introgressed into the B6 background (B6.C3H-*Civq4*; LineB). These lines were generated by repeated backcrosses (10 generations) and selecting progeny at each backcross that retained the *Civq4* locus from the relevant parental line while eliminating the rest of the genome from this parent. Congenic mouse LineB contains approximately 28.48 Mb of the C3H region of *Civq4* (from 31.34 to 59.82 Mb) in the B6 background and congenic mouse LineC contains approximately 23.83 Mb of the B6 region of *Civq4* (from 35.99 to 59.82 Mb) in the C3H background (Figure 4A). Operating under the assumption that the Chr 8 locus mapped in the C3H x B6 cross (Chu et al., 2013) and the WSB × B6 cross are the same, the congenic LineB and LineC (from the C3H and B6 cross) further narrowed the candidate interval by approximately 50%.

Using the parental strains, B6 and C3H, and the reciprocal congenic mouse lines, LineB and LineC, we first analyzed the number of collateral vessel connections between the ACA and MCA. The number of vessel connections of the congenic mouse lines matched that of the parental lines for their overall strain background, and not the smaller congenic interval [B6 (20.4), LineB (21.0), C3H (14.3), LineC (13.5)] (Figures 4B–F). Thus, as expected, the congenic regions did not affect the collateral vessel phenotype. We then measured the effect of the *Civq4* region on ischemic infarct volume. There was no difference in infarct volume between B6 (7.8 mm^3^) and LineB (8.5 mm^3^). Since the B6 strain background exhibits very small cerebral infarcts, we presume that the infarct volume phenotype is near its natural minimum, and this may mask any additional potential neuroprotective effects of the congenic (C3H) locus. By contrast, the infarct volume in LineC animals was 150% larger than C3H animals (38.7 mm^3^ vs. 26.4 mm^3^) after pMCAO (Figures 4G–K). Here, where the B6 congenic region is contributing a risk allele for infarction and tissue damage, the effect of the congenic region (B6) is now apparent. It is important to note that while infarct volume was increased in the LineC animals compared to its background C3H mouse strain, there was no difference in collateral vessel number between the C3H and LineC mice (Figures 4F,K). Therefore, we conclude that the B6 allele in the *Civq4* locus acts as a risk allele for ischemic infarction, and that it modulates infarct volume through a collateral vessel-independent mechanism.

### Coding SNP Variation Identifies Potential Candidate Genes in the Locus

To identify the causal gene(s) modulating infarct volume in the *Civq4* interval, we first surveyed all coding genes in the interval for the presence of non-synonymous coding SNPs (hereafter, coding SNPs). Through this bioinformatic analysis, we identified a total of 964 coding SNPs in 62 coding genes within this interval. Indeed, only 10 genes mapping within the interval did not show any coding SNPs between the mapping strains. Thus, to further refine the candidate gene list, we filtered the gene list by an additional criterion, using the assumption that at least one infarct-modulating gene in the *Civq4* interval is also shared in the overlapping interval mapped between WSB and B6. Therefore, we further required the allele present in the two protective strains (i.e., the C3H and WSB alleles) to differ from the risk strain (i.e., the B6 allele). A total of 85 coding SNPs in 18 coding genes remained (Supplementary Table 2). Next, to determine whether an amino acid substitution within any of these coding SNPs has functional consequences on the respective protein, all the coding SNPs were evaluated using three independent *in silico* prediction algorithms, SIFT, PolyPhen-2, and PROVEAN. Through these *in silico* functional analyses, we found seven genes within *Civq4* that were predicted by at least one algorithm to damage the resulting protein when the B6 allele of the protein was used as the reference (Table 2). However, more detailed analysis of the coding variants in some of the genes in the interval shows that for some gene variants, it is not the C3H and WSB sequence variants, but rather the B6 variants that differ in sequence from the orthologous genes across a wide spectrum of species. This data suggests that the B6 allele at this locus may be functionally damaged, a hypothesis that is consistent (though not obligatory) due to the association of this allele with infarction risk. Thus, we also re-ran the analysis in which we substituted all coding SNPs in the B6 reference amino acid sequences with the C3H and WSB variant and interrogated the predicted effect on protein function when these SNPs were changed to the B6 variant. From this analysis, we found six genes within *Civq4* that were predicted to damage the resulting protein (Table 3). Only one gene, *Msr1*, harbors a coding SNP predicted to be damaging by two algorithms when the coding SNP harbors the C3H and WSB alleles (Table 2). On the other hand, one gene, *Slc7a2*, harbors a coding SNP predicted to be damaging by all three algorithms and another two genes, *Prag1* and *Adam25*, harbor damaging coding SNPs predicted by two algorithms when the coding SNPs harbor the B6 allele (Table 3).

**TABLE 2:** Candidate genes within the 1.5-LOD support interval that have coding SNPs predicted to be damaging for the C3H and WSB alleles.

**Gene**	**Description (Gene synonyms)**	**RS number**	**Coding change**	**Prediction of protein damaging by *in silico* algorithms** **(PolyPhen-2, SIFT, and PROVEAN)**		
				**All three**	**Only two**	**Only one**
*Pragl*	PEAK1 related kinase activating pseudokinase 1 (D8Ertd82e, NACK)	rs47798524	D243Y			P
		rs30475069	R261C			P
*Dlc1*	Deleted in liver cancer 1 (A730069N07Rik, Arhgap7, STARD12, p122-RhoGAP)	rs30480037	L61P			P
*AI429214*	Expressed sequence AI429214	rs30496799	D241E			P
*Msr1*	Macrophage scavenger receptor 1 (MRS-A, MSR-A, SR-AI, SR-AII, Scaral, Scvr)	rs4227116	L93R			S
		rs4227118	K110N			S
		rs4227119	E120A			S
		rs30713937	N202H		P/S	
*Zdhhc2*	Zinc finger, DHHC domain containing 2 (5730415P04Rik, 6430583A19Rik)	rs39259851	S345I			PR
*Adam24*	A disintegrin and metallopeptidase domain 24 (testase 1) (Dtgn5)	rs33077682	A182E			PR
*Adam39*	A disintegrin and metallopeptidase domain 39 (1700056P18Rik)	rs33217945	K466E			S

*Detailed coding SNP information for each gene is available in Supplementary Table 2.*

**TABLE 3:** Candidate genes within the 1.5-LOD support interval that have coding SNPs predicted to be damaging for the B6 allele.

**Gene**	**Description (Gene synonyms)**	**RS number**	**Coding change**	**Prediction of protein damaging by *in silico* algorithms (PolyPhen-2, SIFT, and PROVEAN)**		
				**All three**	**Only two**	**Only one**
*Pragl*	PEAK1 related kinase activating pseudokinase 1 (D8Ertd82e, NACK)	rs30474449	E861G		P/PR	
*Dlc1*	Deleted in liver cancer 1 (A730069N07Rik, Arhgap7, STARD12, p122-RhoGAP)	rs49225660	E192G			P
*AI429214*	Expressed sequence AI429214	rs30495837	S148L			PR
*Micu3*	Mitochondrial calcium uptake family, member 3 (29D0075B16Rik, Efha, Efha2)	rs221623770	L7F			P
*Adam25*	A disintegrin and metallopeptidase domain 25 (testase 2) (Adam25)	rs33291762	H174Q		S/PR	
*Slc7a2*	Solute carrier family 7 member 2 (20.5, Al 158848, Atr, Atrc2, Cat, CAT-2)	rs50403696	A342S	P/S/PR		

*Detailed coding SNP information for each gene is available in Supplementary Table 2.*

### RNA Sequencing Reveals Additional Candidate Genes

**FIGURE 5:**
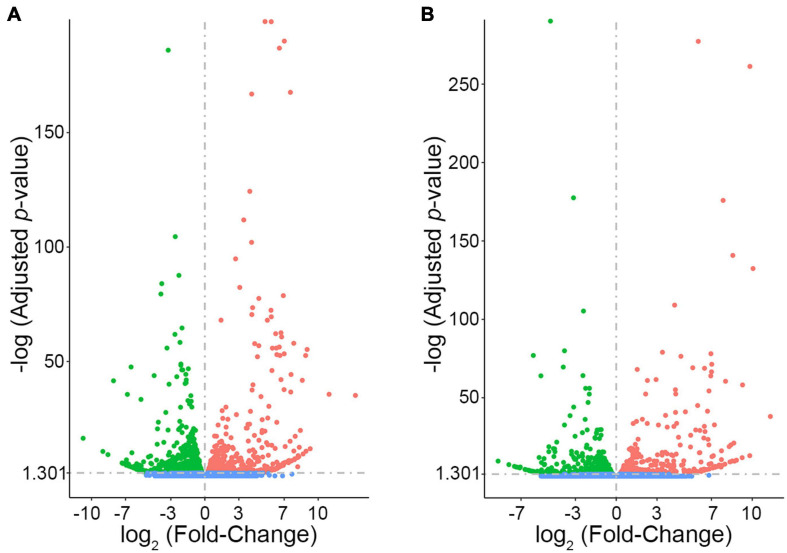
RNA sequencing identifies transcripts showing strain-specific differences in cerebral cortex gene expression. The volcano plots show differential gene expression for either WSB vs. B6 **(A)** or C3H vs. B6 **(B)** from brain cortex determined by RNA sequencing analysis. Each dot represents a different gene with log_2_ fold-changed plotted against log_10_ *p*-value. **(A)** The total number of significantly differentially expressed genes across the mouse genome between WSB and B6 is 1,716 genes, containing 782 upregulated genes indicated by red dots and 934 downregulated genes indicated by green dots, relative to B6. Blue dots (25,956) indicate no significant difference in genes compared between WSB and B6. **(B)** The total number of significantly differentially expressed genes across the mouse genome between C3H and B6 is 2,180 genes, containing 980 upregulated genes indicated by red dots and 1,200 downregulated genes indicated by green dots, relative to B6. Blue dots (25,433) indicate no significant difference in genes compared between C3H and B6.

In order to prioritize additional candidate genes within the locus, we compared transcript levels in cortex tissue between WSB, B6, and C3H adult mice. Differentially expressed genes could be caused by regulatory sequence variation acting in *cis*, and these differences could affect infarct volume. When comparing WSB and B6, a total of 1,716 genes showed a statistically significant difference in transcript levels for genes across the entire mouse genome, and 34 of these were located within the 1.5-LOD support interval on Chr 8 (Figure 5A and Table 4). When comparing C3H and B6, a total of 2,180 genes showed a statistically significant difference in transcript levels among genes across the entire mouse genome, and ten of these were located within the *Civq4* (1.5-LOD support interval) on Chr 8 (Figure 5B and Table 5). Since the two genetic regions identified by independent crosses overlap on Chr 8, we made the assumption that at least one potential infarct-modulating gene would be located within the overlapping region. Using this criterion, the candidate genes that were differentially expressed between WSB and B6 was reduced to only eight genes (highlighted in gray in Table 4). Among these genes only three genes (*Mtmr7*, *Gm5345*, and *Gm40493*) were differentially expressed between WSB vs. B6 and C3H vs. B6. *Gm5345* is annotated as a processed pseudogene and *Gm40493* is a novel, predicted gene without additional annotation. *Mtmr7* is the only of the three genes annotated as protein-coding. We note that all the genes showing different transcript levels within the *Civq4* interval were lower in both C3H and WSB compared to B6 (Tables 4, 5). Details for all of the genes showing differences in transcript levels (1,716 for WSB vs. B6 or 2,180 for C3H vs. B6) are listed in Supplementary Tables 3, 4, respectively.

**TABLE 4:**
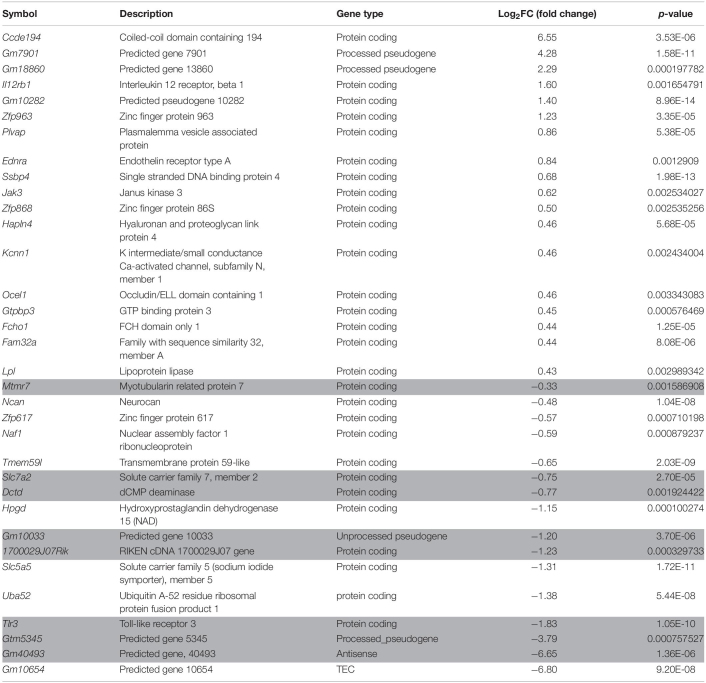
Differential gene expression between WSB and B6 determined by RNA sequencing analysis.

The table lists all genes showing differential expression within the 1.5-LOD support interval (36.16–78.11 Mb) of the F2 mapping between WSB and B6 on Chr 8. Candidate genes are displayed based on fold difference and positive values in the fold change indicate the direction of difference where WSB allele is higher than B6 allele. Genes highlighted with gray color are located in the *Civq4*. Additional information for all 1,716 genes is available in Supplementary Table 3. *TEC, to be experimentally confirmed.

**TABLE 5:** Differential gene expression between C3H and B6 determined by RNA sequencing analysis.

**Symbol**	**Description**	**Gene type**	**Log_2_FC (fold change)**	***p-*value**
*Cnot7*	CCR4-NOT transcription complex, subunit 7	Protein coding	−0.37	0.00227032
*Mtmr7*	Myotubularin related protein 7	Protein coding	−0.38	0.000592403
*Vps37a*	Vacuolar protein sorting 37A	Protein coding	−0.42	0.002065146
*Dlc1*	Deleted in liver cancer 1	Protein coding	−0.48	8.19E-05
*Sorbs2*	Sorbin and SH3 domain containing 2	Protein coding	−0.50	0.000450996
*Micu3*	Mitochondrial calcium uptake family, member 3	Protein coding	−0.60	0.000122213
*Trmt9b*	tRNA methyltransferase 9B	Protein coding	−0.67	0.000345454
*Sgcz*	Sarcoglycan zeta	Protein coding	−1.06	0.003695057
*Gm40493*	Predicted gene, 40493	Anti sense	−4.92	5.94E-05
*Gm5345*	Predicted gene 5345	Processed pseudogene	−6.38	6.39E-06

*The table lists all genes showing differential expression within the 1.5-LOD support interval (*Civq4*; 36.02–50.26 Mb) of the F2 mapping between C3H and B6 on Chr 8. Candidate genes are displayed based on fold difference and negative values in the fold change indicate the direction of difference where the B6 allele is expressed higher than the C3H allele. Additional information for all 2,180 genes is available in Supplementary Table 4.*

## Discussion

In this study, we performed QTL mapping between B6 and WSB mice to identify candidate genes that modulate infarct volume in a collateral vessel-independent manner (i.e., neuroprotection). Since classical inbred mouse strains originate from a relatively limited number of founder haplotypes (Yang et al., 2011), we first surveyed the infarct volume and collateral vessel phenotype in the eight founder strains used in the CC (Lee et al., 2019), which include three wild-derived mouse strains that exhibit much greater genetic diversity that the classical strains. Through this mapping between B6 (classical inbred strain) and WSB (wild-derived strain), we identified a single locus on Chr 8 that reached genome-wide statistical significance and was associated with a difference in infarct size following pMCAO. This locus, located in the interval between 36.16 and 78.11 Mb on Chr 8, overlaps with the neuroprotective locus designated *Civq4* (36.02 and 50.26 Mb) that we previously identified through QTL mapping between C3H and B6 mice (Chu et al., 2013). Similar to our previous study, we found that although B6 mice have smaller infarct volumes compared to WSB mice, the B6 allele at the Chr 8 locus (36.16–78.11 Mb) is associated with larger infarct volumes. Due to the time (>2 years) required to generate a fully congenic strain, we do not have validation data on reciprocal congenic lines between B6 and WSB for the newly mapped Chr 8 locus. However, using reciprocal congenic mouse lines between the C3H and B6 strains, we confirmed that the B6 allele at the *Civq4* locus increases the degree of infarction following pMCAO. Importantly, there was no difference in the number of collateral vessels between the congenic mouse lines and the corresponding background controls. We used a combination of *in silico* SNP prediction and RNA sequencing data analysis to identify candidate genes within the *Civq4* interval. Together, our data demonstrate that the interval from 36.02 to 50.26 Mb on Chr 8 is involved in regulating cerebral infarct volume independently of the collateral vasculature and we have identified a subset of genes within this locus that are viable candidates for modulating infarct volume independently of collateral vessel density.

For this study, we specifically chose to perform QTL mapping using WSB and B6 animals since both of these strains have a high number of collateral vessels. Previous studies have demonstrated that reperfusion of the at-risk tissue following an infarction event by blood flow through existing collateral vessels is strongly associated with a smaller infarct size (Kao et al., 2017). Therefore, by selecting two strains that both have a high number of collateral vessels, and presumably similar levels of reperfusion, we were able to remove the effects of this variable from our analysis to uncover loci associated with neuroprotection.

This study focused on identifying genetic loci involved in neuroprotection. Although neuroprotection may occur as a result of changes within the neurons, changes in other cell types within the brain have also been shown to affect neuronal survival. In fact, previous studies have demonstrated that brain macrophages play a role in regulating outcomes following ischemic stroke (Kim and Cho, 2016). Interestingly, our unbiased, genetic approach identified *Msr1* as a potential candidate gene. This gene encodes for the protein macrophage scavenger receptor 1, which is robustly expressed by macrophages and microglia. Interestingly, other studies have suggested a possible role for *Msr1* in modulating the degree of damage following ischemic stroke by enhancing phagocytic activities in macrophages and microglia to resolve inflammation (Shichita et al., 2017). Therefore, differences in *Msr1* function may underlie some of the observed neuroprotective effects of the *Civq4* locus on Chr 8.

Our study also identified solute carrier family 7 member 2, *Slc7a2*, as a potential candidate gene in modulating the degree of neuroprotection. This protein facilitates transport of cationic amino acids, including L-arginine, across the cell membrane. Interestingly, previous work has demonstrated a role for *Slc7a2* in regulating the activation of macrophages (Yeramian et al., 2006), but the role of *Slc7a2* in brain macrophages following an ischemic event has not been determined. There has also been a limited amount of work investigating the role of *Slc7a2* in ischemic events in organs such as the liver (Ferrigno et al., 2016) and heart (Pan et al., 2011), although these studies did not directly test the role of *Slc7a2* in tissue protection. Given the involvement of macrophages in modulating infarction after stroke (Jian et al., 2019) and the role of *Slc7a2* in L-arginine transport to maintain macrophage activation (Yeramian et al., 2006), *Slc7a2* may indeed have a neuroprotective function following an ischemic event in the brain, warranting further investigation.

Finally, the results from our reciprocal congenic mouse lines support the conclusion that the B6 strain harbors at least one functionally damaged allele in the *Civq4* locus on Chr 8. Specifically, we found that the LineC (C3H.B6-*Civq4*) mice, which have the B6 allele of *Civq4* in the C3H background, have a greater degree of infarction after pMCAO. In addition, some of the genes in this region harbor B6 alleles that differ greatly from the orthologous gene in other species, whereas WSB and C3H strains contain the conserved allele. These data suggest that the B6 background harbors at least one gene variant within the *Civq4* locus that has a detrimental effect on protein function. Currently, the mouse reference genome assembly is derived from the B6 mice, which may give the appearance that all genes in the B6 strain result in wild-type levels of functional protein. However, a recent study (Fontaine and Davis, 2016) has clearly demonstrated that C57BL/6J (B6) strain does in fact harbor null alleles, even when comparing to the closely related C57BL/6N strain. Together, these data demonstrate the importance of assessing the directionality of each parental allele on the phenotype when performing mapping studies, rather than assuming that the B6 allele always represents that functional form of each protein.

## Conclusion

This study has provided further support for the importance of the *Civq4* interval on mouse Chr 8 in neuroprotection following an ischemic stroke. We identified 22 candidate genes mapping within the *Civq4* interval that exhibit either strain-specific coding SNPs (10 candidate genes) (Tables 2, 3) or transcript level differences (15 candidate genes) (Tables 4, 5). Of these 22 genes, three genes, *Dlc1*, *Micu3*, and *Slc7a2*, exhibit both coding and expression level differences. Future studies are required to experimentally test the candidate genes suggested here to determine the contribution of each to neuroprotection, as well as the cell types involved in mediating this protection.

## Data Availability Statement

The original contributions presented in the study are publicly available. This data can be found here: https://www.ncbi.nlm.nih.gov/geo/query/acc.cgi?acc=GSE168383.

## Ethics Statement

The animal study was reviewed and approved by Duke University Institutional Animal Care and Use Committee.

## Author Contributions

HKL and DM designed the research. HKL performed the research. HKL and SW-S analyzed the data. HKL, SW-S, DA, and DM wrote the manuscript. All authors contributed to the article and approved the submitted version.

## Conflict of Interest

The authors declare that the research was conducted in the absence of any commercial or financial relationships that could be construed as a potential conflict of interest.

## Publisher’s Note

All claims expressed in this article are solely those of the authors and do not necessarily represent those of their affiliated organizations, or those of the publisher, the editors and the reviewers. Any product that may be evaluated in this article, or claim that may be made by its manufacturer, is not guaranteed or endorsed by the publisher.

## Footnotes

1
http://cgd.jax.org/mousemapconverter/

2
https://phenome.jax.org/

3
http://genetics.bwh.harvard.edu/pph2/index.shtml

4
http://sift.jcvi.org

5
http://provean.jcvi.org
