# Supplementary material for: A Neuroprotective Locus Modulates Ischemic Stroke Infarction Independent of Collateral Vessel Anatomy
Source: Front Neurosci. 2021 Aug 2;15:705160. doi: 10.3389/fnins.2021.705160 (PMC8366065; doi:10.3389/fnins.2021.705160)
Supplement: Supplementary file 5 [file Presentation_1.pdf]

Supplemental Figures and Tables

A neuroprotective locus modulates ischemic stroke infarction independent of collateral vessel anatomy

Han Kyu Lee[1,*], Sarah E. Wetzel-Strong[1], David L. Aylor[2], Douglas A. Marchuk[1,*]

[1] Department of Molecular Genetics and Microbiology, Duke University Medical Center, Durham, NC, USA

[2] Department of Biological Sciences, North Carolina State University, Raleigh, NC, USA

* Correspondence:

Co-corresponding Authors

Douglas A. Marchuk, douglas.marchuk@duke.edu

Han Kyu Lee, hankyu.lee@duke.edu

Keywords: Ischemic Stroke, Infarct volume, Neuroprotection, Wild-derived mouse strain, Quantitative Trait Locus mapping

Figure S1

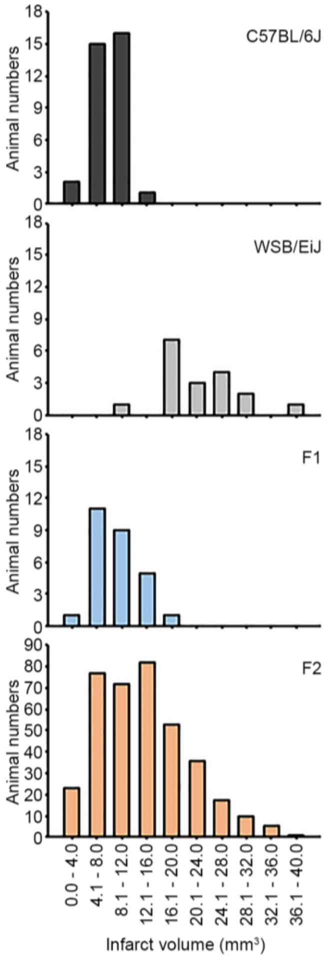

Infarct volume in F2 animals shows relatively wide distribution. Each graph describes the distribution of infarct volume for B6, WSB, F1, F2 animals shown in Figure 1B with scatter plots. The range of the infarct volume is shown on the x-axis, and the y-axis indicates animal numbers.

Figure S2

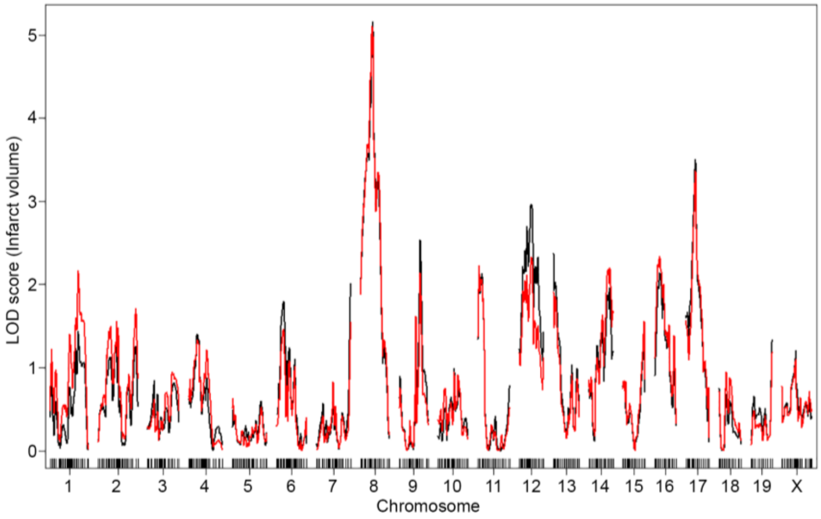

Non-parametric mapping test of the genome-wide QTL mapping analysis. The non-parametric mapping test was performed to validate the genome-wide QTL mapping for infarct volume that was analyzed with the parametric mapping test shown in Figure 2. The non-parametric test indicated by a red line is superimposed on the parametric test indicated by a black line. The two independent mapping plots are nearly identical.

Figure S3

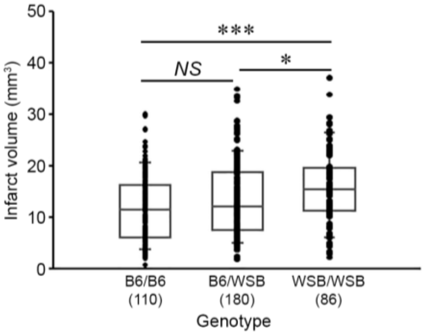

Characterization of the allelic effects of the locus mapping to Chromosome 17. The linkage peak on Chr 17 did not reach a significance threshold ($p < 0.05$) determined by 1,000 permutation test. However, allelic effects on infarct volume of the F2 cohort at gUNC27989312 (LOD score: 3.51) on Chr 17 shows that the B6 allele is protective for infarction. This follows the same trend as the parental strains. Statistial analysis was performed with one-way ANOVA followed by Tukey's multiple comparison test (* $p < 0.05$; *** $p < 0.001$).

Figure S4

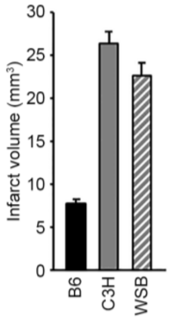

Infarct volume after pMCAO in the three inbred mouse strains used in two independent QTL mapping studies that identified an overlapping interval on Chromosome 8. The same data of infarct volume for each inbred strain are shown in either Fig 1B (for B6 and WSB) or Fig 4K (for B6 and C3H). The graph shows the infarct volume 24 hours after pMCAO. The total number of animals for B6, C3H, and WSB are 34, 24, and 18 animals, respectively. Data represent the mean ± SEM.

Figure S5

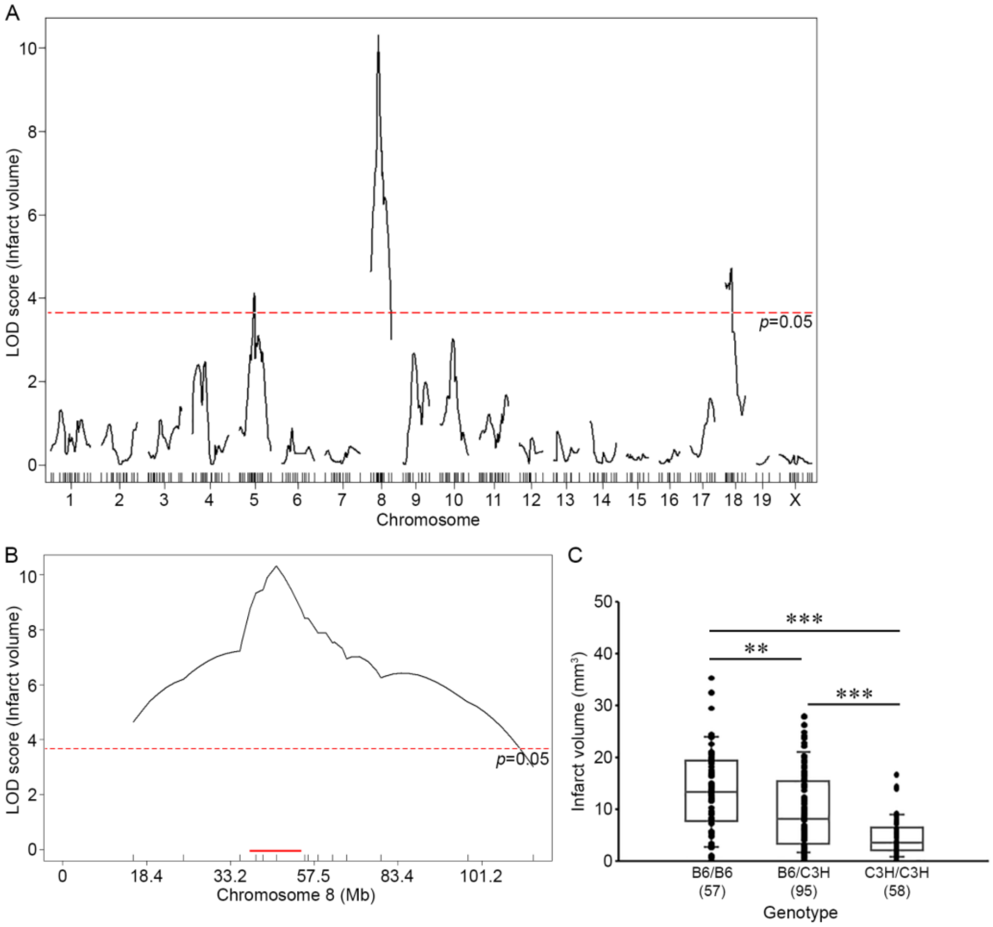

Allelic effects of the Civq4 on Chromosome 8 mapping previously identified in a cross between C3H and B6. (A) The graph presents the re-analysis of a genome-wide QTL mapping scan for infarct volume measured 24 hours after pMCAO using 210 F2 animals (B6 and C3H). Chromosomes 1 through X are represented numerically on the x-axis, and the y-axis represents the LOD score. The significant ($p < 0.05$) level of linkage was determined by 1,000 permutation tests. Three regions of the genome mapping to Chr 5, 8, and 18 display significant QTL mapping to the infarct volume trait with LOD scores of 4.13, 10.31, and 4.71, respectively. **(B)** The graph shows the major locus mapping across Chr 8 using 14 informative SNP markers. The LOD score

at the peak is 10.31 (rs13479735), and the 1.5-LOD support interval is from 36.02 to 50.26 Mb, indicated by the red bar on the graph. **(C)** Allelic effects on infarct volume of the F2 cohort at rs13479735. The B6 allele confers increased susceptibility to infarction and the C3H allele confers protection. Statistical analysis was performed with one-way ANOVA followed by Tukey's multiple comparison test (∗∗ $p < 0.01$; ∗∗∗ $p < 0.001$).

Table S1

Raw data for all Figures. The Excel spreadsheet contains the numerical values with detailed statistical information and array information used to generate all of the figures.

Table S2

Genes mapping within the *Civq4* that harbor coding SNP differences between protective allele and risk allele. The table shows non-synonymous coding SNPs between the protective allele (i.e., C3H and WSB) and the risk allele (i.e., B6). The functional consequences on protein function for each coding SNP was predicted using three independent *in silico* algorithms, SIFT, PolyPhen-2, and PROVEAN. Coding SNP predicted to be either “damaging” or “deleterious” are highlighted in red.

Table S3

Genes show ing strain-specific differential gene expression between WSB and B6. For each of the 1,716 genes, the table displays the *p*-value and fold change, with either a positive value or negative value, corresponding to increased or decreased expression of the WSB allele relative to the B6 allele, respectively.

Table S4

Genes show strain-specific differential gene expression between C3H and B6. For each of the 2,180 genes, the table displays the *p*-value and fold change, with either a positive value or negative value, corresponding to increased or decreased expression of the C3H allele relative to the B6 allele, respectively.
